# Genome-wide identification and characterization of R2R3-MYB genes in *Medicago truncatula*

**DOI:** 10.1590/1678-4685-GMB-2018-0235

**Published:** 2019-11-14

**Authors:** Wei Li, Ying Liu, Jinyue Zhao, Xin Zhen, Changhong Guo, Yongjun Shu

**Affiliations:** 1 College of Life Science and Technology, Harbin Normal University, Harbin Heilongjiang, China.

**Keywords:** Medicago truncatula, MYB, abiotic stress, qRT-PCR

## Abstract

MYB is a large family of plant transcription factors. Its function has been identified in several plants, while there are few reports in *Medicago truncatula*. In this study, we used RNA-seq data to analyze and identify R2R3-MYB genes in the genome of *Medicago truncatula*. Phylogenetic analysis classified 150 MtMYB genes into 21 subfamilies with homologs. Out of the 150 MtMYB genes, 139 were distributed among 8 chromosomes, with tandem duplications (TD) and segment duplications (SD). Microarray data were used for functional analysis of the MtMYB genes during growth and developmental processes providing evidence for a role in tissues differentiation, seed development processes, and especially the nodulation process. Furthermore, we investigated the expression of MtMYB genes in response to abiotic stresses using RNA-seq data, which confirmed the critical roles in signal transduction and regulation processes under abiotic stress. We used quantitative real-time PCR (qRT-PCR) to validate expression profiles. The expression pattern of *M. truncatula* MYB genes under different abiotic stress conditions suggest that some may play a major role in cross-talk among different signal transduction pathways in response to abiotic stresses. Our study will serve as a foundation for future research into the molecular function of *M. truncatula* R2R3-MYB genes.

## Introduction

Myeloblastosis (MYB) genes are one of the largest transcription factors families in the plant kingdom (Romero *et al.*, 1998; Rosinski and Atchley, 1998). MYB typically contain an MYB-binding domain at the N-terminus, composed of 1-4 imperfect repeats, with approximately 51-52 amino acid residues of incomplete conserved peptides encoding three α-helices (Kanei-Ishii *et al.*, 1990; Lipsick, 1996; Stracke *et al.*, 2001). These three α-helices form a helix-turn-helix (HTH) structure and fold with three relatively conserved tryptophan residues, separated by 18-19 amino acid residues of regular arrangement, and further participate in the formation of hydrophobic interactions (Ogata *et al.*, 1995). The structure of the MYB domain reveals that the HTH interacts with the major groove of DNA (Ogata *et al.*, 1994). MYB-containing genes have a diverse number of MYB proteins containing incomplete repeats and are divided into four categories, 1R-MYB, R2R3-MYB, R1R2R3-MYB, and 4R-MYB (Jin and Martin, 1999).

In the MYB gene family, R2R3-MYB is the largest category in plants and yeast (Martin and Pazares, 1997; Jin and Martin, 1999). To date, 126 R2R3-MYB genes have been identified in *Arabidopsis* (Stracke *et al.*, 2001), 157 in maize (Du *et al.*, 2012a), 244 in soybean (Du *et al.*, 2012b), 205 in *Gossypium raimondii* (He *et al.*, 2016), and 166 in cassava (Liao *et al.*, 2016). R2R3-MYB transcription factors are involved with abiotic stress response, reactive oxygen species signaling pathways, secondary metabolism, and hormone signaling pathways. AtMYB41 from *Arabidopsis* is induced in response to high salinity, drought, cold, and abscisic acid (Lippold *et al.*, 2009). AtMYB102 are rapidly induced by osmotic stress and abscisic acid (ABA) treatment (Denekamp and Smeekens, 2003). OsMYB55 is induced in rice by high temperature and over-expression of OsMYB55 resulted in improved plant growth under high temperature (Elkereamy *et al.*, 2012). Meanwhile, OsMYB91 plays a role in plant growth regulation and salt stress tolerance in rice (Zhu *et al.*, 2015). TaMYB4 of wheat is induced by salicylic acid, ethylene, abscisic acid, and methyl jasmonate, demonstrating a role of TaMYB4 in response to biotic stress (Al-Attala *et al.*, 2014). GbMYB5 from *Gossypium barbadense* is positively involved in response to drought stress during plant development (Chen *et al.*, 2015). GbMYBFL, involving in flavonoid biosynthesis, is most closely related to R2R3-MYB and displays high similarity to MYB from other plants (Zhang *et al.*, 2017).

*Medicago truncatula* is a model plant for genetic research of legume plants (Young and Udvardi, 2009), as it has a small genome, high genetic transformation efficiency, self-pollination, nitrogen fixing, along with high biological diversity. Crop improvement of legume breeding has become an important subject (Lee *et al.*, 2017). MtERF and MtMAPKKK gene families of *M. truncatula* are valuable for characterizing molecular function to improve stress tolerance in plants (Li *et al.*, 2016; Shu *et al*.. 2016). However, the R2R3-MYB family is poorly identified on a genomic level in *M. truncatula*.

In this study, we performed a genome-wide analysis of the R2R3-MYB gene family in *M. truncatula*, including multiple alignment analysis, phylogenetic analysis, chromosome localization, gene duplication analysis, and expression profiling. We used quantitative real-time reverse transcription (qRT-PCR) experiments to compare and analyze five stress treatments (ABA, cold, freezing, drought, and salt) with a control treatment. Our study will serve as a foundation for future research into the molecular function of *M. truncatula* R2R3-MYB genes.

## Materials and Methods

### Plant growth and treatment

*M. truncatula* (cv. JemalongA17) seeds were grown in a growth chamber (Conviron E15), between 18ºC (night) and 24ºC (day), with humidity ranging from 60% to 80%, and a 14/10 h light-dark period (light, 06:00–20:00). The seedlings were irrigated with half-strength Hoagland solution once every other day. At 8 weeks, *M. truncatula* were randomly divided into six groups for stress treatments. Plants treated with cold stress (B group) and freezing stress (C group) were transferred to chambers set at 4 ºC and -8 ºC, respectively. Plants treated with drought stress (D group) and salt stress (E group) were respectively treated with 300 mM mannitol and 200 mM NaCl solutions. Plants in the ABA (F group) treatment group were sprayed with 100 μM ABA solution. The control (untreated, A group) and treated (B-F groups) seedlings were harvested 3 h after treatment. For each group, five randomly chosen whole seedlings were pooled to form a biological replicate. All plant samples were frozen in liquid nitrogen and stored at -80 ºC until use.

### Database search for MYB proteins in *M. truncatula*

*M. truncatula* genome and protein sequences were downloaded from JCVI (http://jcvi.org/medicago/, Mt4.0) (Young *et al.*, 2011). R2R3-MYB protein sequences of *Arabidopsis* were obtained from TAIR (http://www.arabidopsis.org/) (Poole, 2007) and served as queries to search against the *M. truncatula* proteins using the BLASTP program with e-values of 1E-3. All BLAST hits were retrieved and searched using the Hidden Markov Model (HMM) profile of the R2R2-MYB DNA-binding domain (PF000249), which was downloaded from the Pfam website (http://pfam.xfam.org/) (Finn *et al.*, 2014).

### Phylogenetic analysis of the *M. truncatula* R2R3-MYB gene family

Phylogenetic analysis was performed using MEGA (Version 5.0), and constructed using neighbor-joining (NJ) methods. The NJ method used the following parameters: Poisson correction, pair-wise deletion, and 1000 bootstrap analysis for statistical reliability (Tamura *et al.*, 2011).

### Chromosomal location and gene duplication of R2R3-MYB gene family

The location of R2R3-MYB genes were mapped to different chromosomes using the Circos software (http://circos.ca/) (Krzywinski *et al.*, 2009). If two genes with similarities of more than 85% were separated by four or fewer gene loci, they were identified as tandem duplications (TD). Otherwise, they were classified as segmental duplications (SD). The duplications with R2R3-MYB genes were identified by plant genome duplications (PGDD, http://chibba.agtec.uga.edu/duplication/) (Lee *et al.*, 2012, Shu *et al.*, 2016), and duplicated genes between different chromosomes or loci were linked in the diagrams.

### Expression analysis of R2R3-MYB genes in plant growth and development using high throughput data

We studied the expression analysis of R2R3-MYB genes during growth and development and under different abiotic stress treatments. Gene expression data were downloaded from the *Medicago truncatula* Gene Expression Atlas (MtGEA) Project (MtGEA, http://mtgea.noble.org/v3/) (Benedito *et al.*, 2008). Genome-wide transcriptome data from *M. truncatula* in different tissues during development were downloaded from the NCBI short read archive database (SRA database) (http://www.ncbi.nlm.nih.gov).

### Expression analysis of R2R3-MYB genes under different abiotic stress treatment conditions

Under six different abiotic stress treatments, MtMYB gene expressional values were evaluated using the TopHat (Trapnell *et al.*, 2009) and Cufflinks (Trapnell *et al.*, 2012) software. The data were analyzed, clustered, and displayed using the ggplot2 of R software (Version 3.1.0), as our previous research described (Shu *et al.*, 2016).

### Quantitative reverse transcription PCR (qRT-PCR) analysis of R2R3-MYB genes expressed in *M. truncatula*

Ten R2R3-MYB genes were selected and quantitative primers were designed based on genes sequences, while the ACTIN and GAPDH genes served as reference genes (see Table S1). Total RNA was extracted from *M. truncatula* grown under the six conditions (control, ABA, drought, salt, cold, and freezing) using a total RNA kit (Tiangen, Beijing, China) according to the manufacturer’s instructions. The RNA extracts were reverse transcribed to cDNA, using a PrimeScript RT reagent Kit (Toyobo, Shanghai, China). qRT-PCR was performed using the LightCycler^®^96 System (Roche, Rotkreuz, Switzerland) with SYBR Premix Ex TaqTM II (Toyobo, Shanghai, China). The experiments were repeated for three biological replicates and the PCR conditions were set as follows: 95 ºC for 2 min, 40 cycles of 95 ºC for 30 s, 55 ºC for 30 s, and 72 º for 1 min. The fold change value was calculated using the expression abundances, which based on the 2^-^ΔΔCT method.

## Results

### Identification and classification of R2R3-MYB genes in *M. truncatula*

To identify R2R3-MYB genes in *M. truncatula*, *Arabidopsis* R2R3-MYB gene sequences were used as queries with e-value set as 1E-3. The gene name, gene locus, chromosome location, amino acid sequence, introns, family groups, and isoelectric point (pI) are described in Table 1. Out of the 150 MtMYB genes, 139 genes were distributed across 8 chromosomes of *M. truncatula*. The remaining 11 MtMYB genes were MtMYB5, 6, 7, 13, 14, 16, 34, 35, 36, 37, and 52. The length of these 139 R2R3-MYB ranged from 194 to 1514 amino acids, with an intron distribution of 0-12, and the isoelectric point were distributed from 4.77 to 9.93.

**Table 1:** List of all MtMYB genes identified in the *Medicago truncatula* genome.

Gene Name	Gene Locus	Chromosome Location	AA	Introns	Family Group	pI
MtMYB001	Medtr3g039990	chr3:13992780-13994617	312	2	1	5.44
MtMYB002	Medtr6g012180	chr6:3615718-3617850	319	2	1	5.76
MtMYB003	Medtr7g010210	chr7:2460324-2462646	272	3	1	8.94
MtMYB004	Medtr7g087130	chr7:33927709-33929377	339	2	1	6.54
MtMYB005	Medtr0140s0030	scaffold0140:9682-11671	335	2	1	6.6
MtMYB006	Medtr0489s0020	scaffold0489:9594-10964	335	2	1	6.6
MtMYB007	Medtr0251s0050	scaffold0251:9249-10577	273	1	2	5.09
MtMYB008	Medtr1g043050	chr1:16126632-16128228	255	2	2	5.58
MtMYB009	Medtr1g043080	chr1:16137369-16139502	256	2	2	5.6
MtMYB010	Medtr1g076150	chr1:33730166-33733155	259	2	2	5.31
MtMYB011	Medtr7g096930	chr7:38915313-38918018	279	2	2	5.35
MtMYB012	Medtr7g115650	chr7:47812651-47813895	208	2	2	8.51
MtMYB013	Medtr0008s0390	scaffold0008:236494-237588	273	1	2	5.55
MtMYB014	Medtr0008s0470	scaffold0008:261364-262450	292	1	2	5.34
MtMYB015	Medtr2g067420	chr2:28203900-28207822	330	2	3	4.74
MtMYB016	Medtr0063s0090	scaffold0063:54059-56064	224	2	3	5.78
MtMYB017	Medtr4g073420	chr4:27807701-27809440	286	1	4	8.65
MtMYB018	Medtr4g485530	chr4:33337240-33340075	194	2	4	9.68
MtMYB019	Medtr5g079670	chr5:34066783-34068495	214	2	4	9.14
MtMYB020	Medtr8g095390	chr8:39916215-39917940	316	2	4	9.14
MtMYB021	Medtr4g100720	chr4:41544004-41544940	176	3	5	8.25
MtMYB022	Medtr4g125520	chr4:52070924-52072242	308	2	5	8.34
MtMYB023	Medtr8g020490	chr8:7194946-7196540	255	2	5	8.55
MtMYB024	Medtr5g078800	chr5:33679342-33680505	266	2	6	8.76
MtMYB025	Medtr5g078860	chr5:33697936-33699039	307	2	6	8.76
MtMYB026	Medtr5g078910	chr5:33733725-33736557	301	2	6	9.06
MtMYB027	Medtr5g078930	chr5:33748446-33750303	220	2	6	9.74
MtMYB028	Medtr5g078950	chr5:33762292-33763827	266	2	6	9.14
MtMYB029	Medtr5g079120	chr5:33821199-33824299	307	3	6	9.12
MtMYB030	Medtr5g079220	chr5:33865422-33867467	277	2	6	8.08
MtMYB031	Medtr5g079290	chr5:33903955-33904999	265	2	6	9.11
MtMYB032	Medtr7g017260	chr7:5468456-5471716	276	2	6	7.59
MtMYB033	Medtr8g060940	chr8:21305461-21306435	245	2	6	5.83
MtMYB034	Medtr0001s0360	scaffold0001:148343-149371	254	2	6	8.13
MtMYB035	Medtr0193s0090	scaffold0193:23279-25596	208	2	6	9.27
MtMYB036	Medtr0197s0010	scaffold0197:3158-6293	230	2	6	5.79
MtMYB037	Medtr0247s0040	scaffold0247:19831-22448	180	2	6	9.56
MtMYB038	Medtr2g034790	chr2:13342703-13345610	333	2	7	5.57
MtMYB039	Medtr4g121460	chr4:50199327-50201231	340	2	7	5.21
MtMYB040	Medtr7g117730	chr7:48891947-48893156	257	1	7	9.19
MtMYB041	Medtr8g027345	chr8:9621235-9622899	301	1	7	7.03
MtMYB042	Medtr1g100653	chr1:45593395-45595446	296	2	9	7.16
MtMYB043	Medtr4g082230	chr4:31939369-31942857	408	2	9	6.02
MtMYB044	Medtr4g082290	chr4:31999665-32001816	408	2	9	5.77
MtMYB045	Medtr6g012690	chr6:3910120-3912657	358	2	9	5.54
MtMYB046	Medtr7g011170	chr7:2948411-2950937	310	2	9	6.57
MtMYB047	Medtr7g076740	chr7:28944308-28947368	305	2	9	8.15
MtMYB048	Medtr8g031360	chr8:11745382-11748030	298	2	9	5.92
MtMYB049	Medtr4g478180	chr4:30006885-30009067	354	3	10	6.36
MtMYB050	Medtr5g007370	chr5:1317895-1319526	300	1	10	6.25
MtMYB051	Medtr5g029840	chr5:12551795-12553088	335	1	10	6.26
MtMYB052	Medtr0008s0280	scaffold0008:117749-119885	232	2	10	6.21
MtMYB053	Medtr2g011660	chr2:2891380-2892991	346	2	11	5.85
MtMYB054	Medtr2g089450	chr2:37807548-37809745	251	2	11	7.62
MtMYB055	Medtr2g089620	chr2:37880385-37882262	327	2	11	6.45
MtMYB056	Medtr4g091490	chr4:36245993-36247326	359	2	11	5.87
MtMYB057	Medtr1g085040	chr1:37962367-37964302	370	2	13	7.1
MtMYB058	Medtr1g085640	chr1:38268101-38269978	389	2	13	6.1
MtMYB059	Medtr1g112760	chr1:51044088-51045093	231	2	13	8.61
MtMYB060	Medtr3g077110	chr3:34625609-34627040	350	2	13	5.71
MtMYB061	Medtr4g105130	chr4:43559175-43561153	362	2	13	5.51
MtMYB062	Medtr7g110830	chr7:45437545-45439504	368	2	13	5.28
MtMYB063	Medtr7g111290	chr7:45674622-45676610	382	2	13	6.26
MtMYB064	Medtr1g017000	chr1:4609270-4612231	253	2	14	6.22
MtMYB065	Medtr1g017140	chr1:4685126-4687287	342	2	14	7.21
MtMYB066	Medtr2g095520	chr2:40818577-40820355	321	2	14	7.02
MtMYB067	Medtr3g103570	chr3:47845667-47847265	348	2	14	6.26
MtMYB068	Medtr4g057635	chr4:21173419-21175385	325	2	14	6.67
MtMYB069	Medtr4g097570	chr4:40253351-40254860	294	2	14	8.04
MtMYB070	Medtr4g128670	chr4:53556826-53558636	351	2	14	5.43
MtMYB071	Medtr5g014990	chr5:5113035-5114677	310	2	14	7.49
MtMYB072	Medtr6g090405	chr6:34357171-34358762	256	2	14	5.53
MtMYB073	Medtr7g102110	chr7:41207062-41209098	273	2	14	6.13
MtMYB074	Medtr1g100667	chr1:45604161-45606367	273	2	16	9.4
MtMYB075	Medtr6g055910	chr6:20067285-20071069	284	2	16	6.55
MtMYB076	Medtr1g083630	chr1:37214098-37220601	631	10	18	8.87
MtMYB077	Medtr1g085770	chr1:38342281-38444471	308	3	18	7.64
MtMYB078	Medtr1g085880	chr1:38446474-38447221	194	1	18	9.25
MtMYB079	Medtr2g088170	chr2:37135155-37138193	460	2	18	5.04
MtMYB080	Medtr5g062790	chr5:26032596-26033729	305	2	18	4.67
MtMYB081	Medtr5g088080	chr5:38180518-38181517	267	2	18	5.06
MtMYB082	Medtr5g088150	chr5:38189614-38190613	267	2	18	5.06
MtMYB083	Medtr5g088610	chr5:38446993-38448346	356	2	18	5.03
MtMYB084	Medtr5g088640	chr5:38455878-38456905	276	2	18	5.06
MtMYB085	Medtr5g488170	chr5:38207742-38208675	245	2	18	8.27
MtMYB086	Medtr5g488210	chr5:38216839-38217838	267	2	18	5.06
MtMYB087	Medtr6g006030	chr6:968935-973160	210	2	19	6.79
MtMYB088	Medtr7g035075	chr7:13292852-13298285	209	2	19	6.07
MtMYB089	Medtr1g086510	chr1:38706430-38707982	266	2	20	6.71
MtMYB090	Medtr1g086530	chr1:38719605-38720569	279	1	20	5.57
MtMYB091	Medtr1g110460	chr1:49847994-49849320	312	2	20	6.97
MtMYB092	Medtr2g033170	chr2:12558787-12561118	330	2	20	5.54
MtMYB093	Medtr4g123040	chr4:50776703-50778712	316	2	20	6.9
MtMYB094	Medtr4g082040	chr4:31789875-31791702	428	2	21	5.48
MtMYB095	Medtr5g041570	chr5:18244022-18246575	439	2	21	5.81
MtMYB096	Medtr6g027340	chr6:9347619-9348708	206	2	21	9.43
MtMYB097	Medtr6g027370	chr6:9359727-9361707	353	2	21	9.13
MtMYB098	Medtr6g074860	chr6:27791872-27795423	242	2	21	8.94
MtMYB099	Medtr7g086960	chr7:33824449-33826853	357	2	21	8.87
MtMYB100	Medtr3g101290	chr3:46598679-46599616	227	0	22	6.39
MtMYB101	Medtr4g094982	chr4:39426938-39428438	272	0	22	5.76
MtMYB102	Medtr5g016510	chr5:5923364-5924314	317	0	22	8.35
MtMYB103	Medtr5g082910	chr5:35747490-35748793	289	1	22	8.33
MtMYB104	Medtr6g015455	chr6:5126272-5138191	1514	9	22	6.6
MtMYB105	Medtr4g019370	chr4:6040082-6046125	351	1	23	6.1
MtMYB106	Medtr3g074520	chr3:33695057-33696802	347	2	24	5.76
MtMYB107	Medtr1g026870	chr1:8820780-8826887	984	10	25	5.45
MtMYB108	Medtr1g045610	chr1:17117606-17119422	259	2	25	9.28
MtMYB109	Medtr1g057980	chr1:25521596-25523733	298	2	25	6.68
MtMYB110	Medtr1g062940	chr1:27632125-27633748	219	3	25	5.7
MtMYB111	Medtr1g073170	chr1:32447073-32448262	258	2	25	7.64
MtMYB112	Medtr2g064160	chr2:27171680-27173646	314	2	25	6.14
MtMYB113	Medtr2g096380	chr2:41173925-41175393	237	2	25	6.2
MtMYB114	Medtr2g097910	chr2:41830130-41832026	320	1	25	5.63
MtMYB115	Medtr2g099740	chr2:42787607-42789410	402	1	25	6.27
MtMYB116	Medtr3g065440	chr3:29570010-29573532	312	3	25	4.81
MtMYB117	Medtr3g077650	chr3:34915543-34917386	448	2	25	6.65
MtMYB118	Medtr3g083540	chr3:37705148-37706353	304	1	25	6.83
MtMYB119	Medtr3g097450	chr3:44673726-44675570	298	2	25	8.71
MtMYB120	Medtr3g110028	chr3:50889343-50895792	904	11	25	5.08
MtMYB121	Medtr3g461490	chr3:24436965-24438138	326	2	25	6.31
MtMYB122	Medtr4g063100	chr4:23343932-23345888	254	2	25	5.44
MtMYB123	Medtr4g065017	chr4:24357511-24360058	333	1	25	5.31
MtMYB124	Medtr4g088015	chr4:34653348-34657396	443	2	25	9.15
MtMYB125	Medtr4g102380	chr4:42421826-42423447	273	1	25	5.26
MtMYB126	Medtr4g105660	chr4:43865250-43867027	431	2	25	6.39
MtMYB127	Medtr5g010020	chr5:2590454-2592700	340	2	25	5.8
MtMYB128	Medtr5g010650	chr5:2899487-2902910	477	7	25	8.32
MtMYB129	Medtr5g038910	chr5:17123381-17129369	391	10	25	6.19
MtMYB130	Medtr5g049190	chr5:21549233-21551259	243	2	25	6.36
MtMYB131	Medtr5g070020	chr5:29670807-29672960	356	2	25	8.58
MtMYB132	Medtr5g078140	chr5:33378172-33380732	321	2	25	5.12
MtMYB133	Medtr6g009430	chr6:2790201-2792486	337	2	25	5.59
MtMYB134	Medtr7g035350	chr7:13467452-13470094	451	2	25	5.66
MtMYB135	Medtr7g037130	chr7:13641861-13644153	359	2	25	8.62
MtMYB136	Medtr7g037260	chr7:13704241-13706602	359	2	25	8.99
MtMYB137	Medtr7g061330	chr7:22157000-22164437	531	7	25	9.15
MtMYB138	Medtr7g061550	chr7:22184367-22187445	360	1	25	9.39
MtMYB139	Medtr7g450950	chr7:17066918-17069310	339	2	25	5.53
MtMYB140	Medtr7g451170	chr7:17173579-17176966	413	2	25	9.22
MtMYB141	Medtr7g461410	chr7:22216202-22220641	567	6	25	8.75
MtMYB142	Medtr8g006470	chr8:571945-573793	288	2	25	6.22
MtMYB143	Medtr8g017340	chr8:5841430-5843448	493	2	25	4.92
MtMYB144	Medtr8g017350	chr8:5847584-5850190	644	3	25	4.79
MtMYB145	Medtr8g017390	chr8:5885779-5887719	476	2	25	5.34
MtMYB146	Medtr8g017440	chr8:5919956-5921521	342	2	25	6.45
MtMYB147	Medtr8g017500	chr8:5958601-5960679	349	2	25	5.96
MtMYB148	Medtr8g017540	chr8:5983119-5985752	395	3	25	6.07
MtMYB149	Medtr8g098860	chr8:41349431-41351751	321	2	25	5.85
MtMYB150	Medtr8g468380	chr8:24758342-24766275	437	12	25	8.72

### Phylogenetic analysis of the R2R3-MYB genes in *M. truncatula*

**Figure 1:**
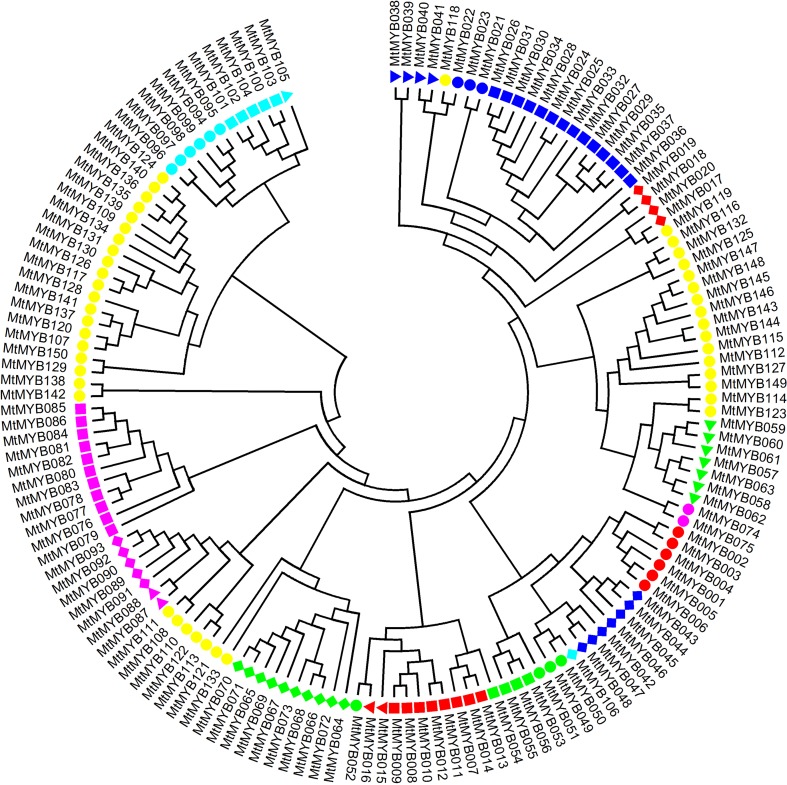
Phylogenetic tree analysis of the R2R3-MYB transcription factors in *Medicago truncatula*. Red circle: subgroup 1; red square: subgroup 2; red triangle: subgroup 3; red diamond: subgroup 4; blue circle: subgroup five; blue square subgroup six; blue indicates subgroup seven; blue diamond subgroup nine; green circular subgroup ten; green square subgroup 11; green triangle: subgroup 13; green diamond: subgroup 14; pink circle: subgroup 16; pink square: subgroup 18; pink triangle: subgroup 19; pink diamond: subgroup 20; cyan circle: subgroup 21; cyan square: subgroup 22; cyan triangle: subgroup 23; cyan diamond: subgroup 24; yellow circle: subgroup 25.

To investigate the evolutionary relationships of R2R3-MYB genes, we performed multiple sequence alignment and phylogenetic analysis. MtMYB transcription factors were queried against AtMYB transcription factors using BLASTP, and we constructed phylogenetic trees of the MtMYB transcription factor family using ClustalW2 and MEGA. MtMYB genes were divided into 21 subfamilies (Figure 1) consistent with the distribution of AtMYB gene family members. The subfamily 1 has six members, subfamily 6 has 14 members, subfamily 13 has seven members, subfamily 14 has ten members, and subfamily 18 has eleven members. This is similar to the distribution of the *Arabidopsis* subfamily members. R2R3-MYB transcription factors appeared to be conserved in plants as the evolution of *M. truncatula* is present in the evolution of *Arabidopsis*.

### Chromosomal location and gene duplication of R2R3-MYB gene family

**Figure 2:**
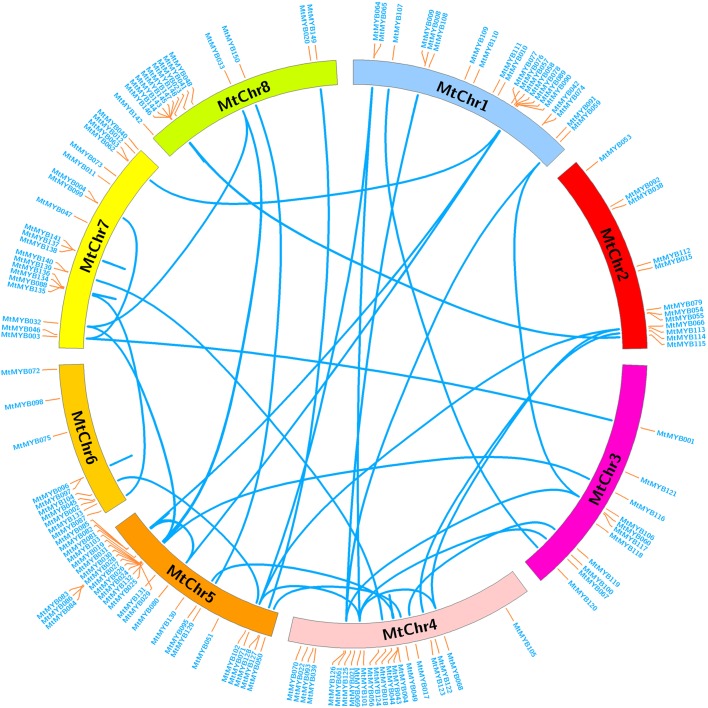
Chromosome distribution and expansion analysis of R2R3-MYB transcription factors in *Medicago truncatula.* The genome locations of R2R3-MYB transcription factors were retrieved from Medicago genome website, and the duplications between R2R3-MYB genes identified using software PGDD and BLAST analysis.

To determine the evolution and expansion of MYB genes, we used Circos software to construct the distribution of MYB genes across chromosomes (Figure 2). Out of the 150 R2R3-MYB genes, 139 were distributed across 8 chromosomes, mainly chromosome 1, 4, 5, and 7 (MtChr1, MtChr4, MtChr5 and MtChr7, respectively). There were 28 R2R3-MYB genes located on MtChr5 alone. The fewest number of genes were found on MtChr6 with ten R2R3-MYB genes. Using sequence alignment, mainly through gene duplication, 128 out of 139 genes were duplicated and divided into two categories. There were 68 segment duplications (SD) caused by the amplification of R2R3-MYB transcription factor members on different chromosomes and 60 tandem duplications (TD) resulting from the generation of R2R3-MYB transcription factor gene clusters. The SD and TD genes were mainly found on MtChr4, MtChr5, MtChr6, MtChr7, and MtChr8, while other chromosomes only contained SD genes. The regions containing TD genes were hot regions of gene distribution. These duplications may have led to the expansion of the MtMYB gene family in the *M. truncatula* genome.

### Expression analysis of R2R3-MYB genes in growth and development

**Figure 3:**
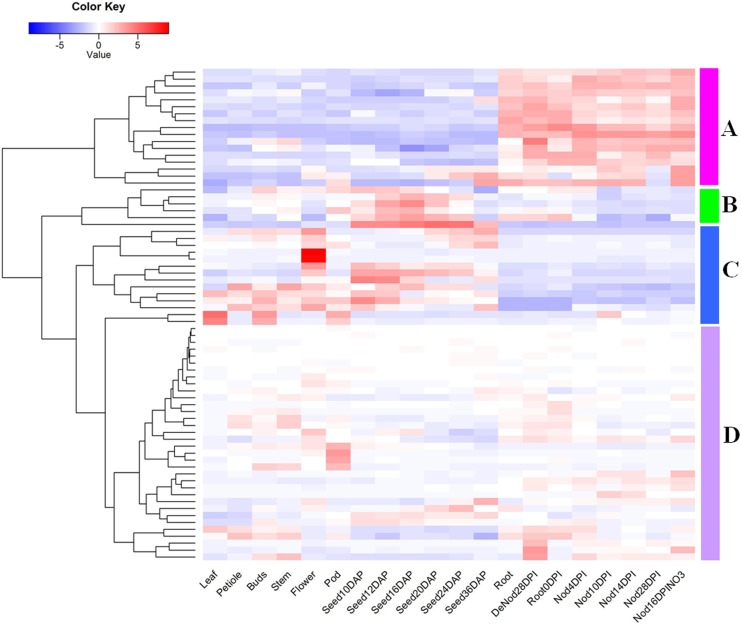
Microarray expression data of R2R3-MYB transcription factors in *Medicago truncatula*. The heatmap was generated using R gplots package. The expressional values of 71 R2R3-MYB genes were retrieved from MtGEA, and they were normalized and used as input, red represents high expressional levels, while blue represents low expressional level.

The expression information of 71 R2R3-MYB transcription factors were extracted and analyzed by cluster analysis (Figure 3 and Table S2) using the annotation information of microarray data based on MtGEA. We clustered the 71 R2R3-MYB transcription factors into four groups (A-D). Group A was mainly concentrated in the roots and nodules of *M. truncatula*. There was only low expression in other tissues and during developmental processes. Group B was mainly expressed in the development of seed. Group C genes were expressed in various organ tissues. These results indicated that they were both involved in tissues construction process of *M. truncatula*. Finally, Group D genes were expressed at a low level in various tissues and during developmental processes.

**Figure 4:**
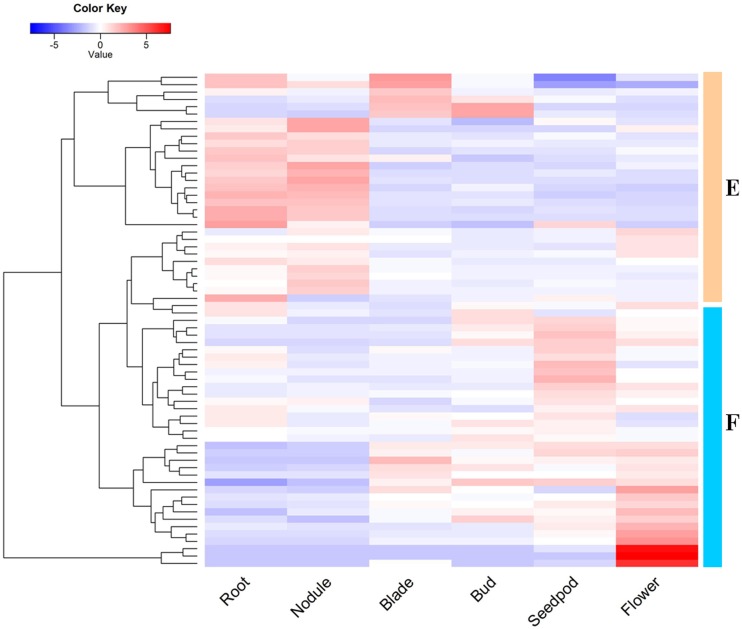
RNA-seq analysis of R2R3-MYB transcription factors in *Medicago truncatula*. The heatmap was generated using R gplots package, and the FPKM values of *Medicago truncatula* genes were evaluated and normalized based RNA-seq data from NCBI SRA database. The plot data included expressional profiles of 67 R2R3-MYB genes in six tissues, and red represents high expressional levels, while blue represents low expressional level.

The transcriptome sequencing data of *M. truncatula* were downloaded from the NCBI SRA database, and the gene expression level was obtained using TopHat2 and Cufflinks analyses. We extracted 67 R2R3-MYB transcription factors (Figure 4 and Table S3). Multiple genes with high expression in the roots and nodules were classified into Group E, while Group F contained numbers of paralog genes, which were mainly up-regulated in the flower, the carps, and inflorescence.

### Expression analysis of R2R3-MYB genes under different abiotic stress treatment conditions

**Figure 5:**
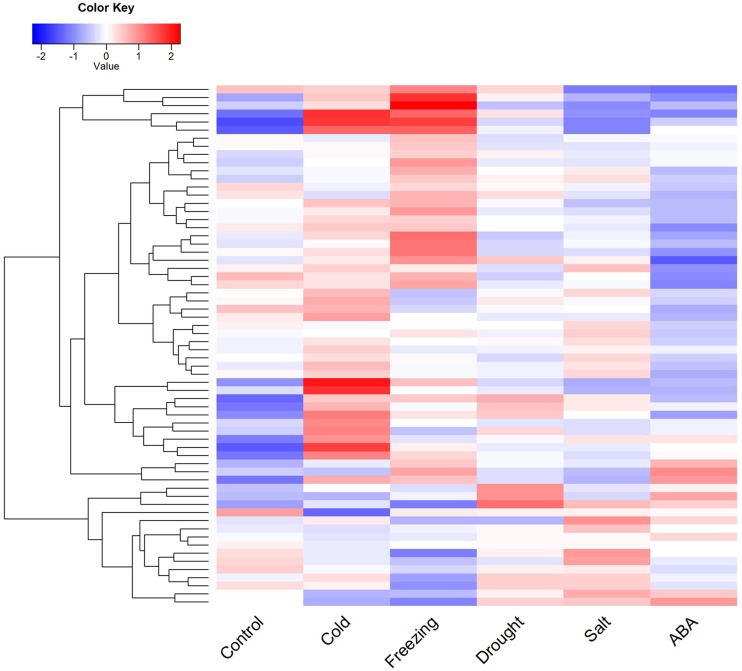
Expression profile analysis of R2R3-MYB transcription factors in response to abiotic stresses in *Medicago truncatula*. The heatmap was generated using R gplots package, and the FPKM values of *Medicago truncatula* genes were evaluated and normalized based RNA-seq data from NCBI SRA database. The plot data included expressional profiles of 64 R2R3-MYB genes in response to abiotic stress, and red represents high expressional levels, while blue represents low expressional level.

RNA-seq was used to investigate the expression of R2R3-MYB transcription factors in response to abiotic stress of *M. truncatula*. Out of the 150 MtMYB genes, 64 were expressed in response to abiotic stress (cold, freezing, drought, salt and ABA) (Figure 5 and Table S4). In the control treatment, multiple genes were down-regulated or showed no change in expression, yet few genes were up-regulated. We compared genes regulated in response to abiotic stress to the control treatment and found genes highly up-regulated in response to cold and freezing stresses (50/64, 78.1%). These genes interacted with their paralog genes showing co-expression. In response to drought, high salt, and ABA stresses, multiple genes showed opposing expression and co-expression of the pairs of paralog genes. Significantly, most of these genes showed only low expression or no expression. In particular, some genes were down-regulated and co-expressed in response to ABA stress. It was suggested that the expression of these transcription factors are induced by ABA hormones, which regulate the downstream response genes and affect the response of plants to drought stress and salt stress.

qRT-PCR validation of R2R3-MYB genes expression

**Figure 6:**
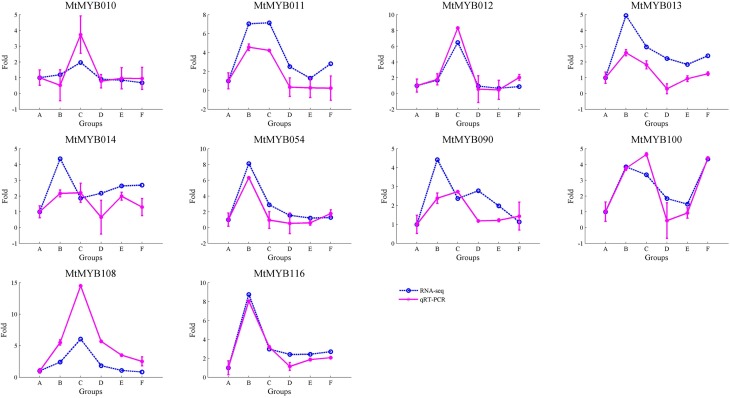
qRT-PCR validation of R2R3-MYB transcription factors in *Medicago truncatula*. All expressional levels of each R2R3-MYB genes were normalized with control expression value (group A) set as one. The groups B (cold stress), C (freezing stress), D (drought stress), E (salt stress), and F (ABA treatment) were compared with control group, and fold-changes were calculated. The fold-change data was used to create the plot, and the blue indicates RNA-seq results, while pink indicates qRT-PCR analysis results.

To validate the reliability of the RNA-seq data under abiotic stress in *M. truncatula*, we selected 10 R2R3-MYB transcription factors (MtMYB010, MtMYB011, MtMYB012, MtMYB013, MtMYB014, MtMYB054, MtMYB090, MtMYB100, MtMYB108, and MtMYB116) from *M. truncatula* for qRT-PCR validation. The expression levels of the RNA-seq data and qRT-PCR gene expression analysis resulted in a correlation coefficient of 0.85 (Figure 6). These results suggest the RNA-seq data are highly reliable.

## Discussion

To date, R2R3-MYB transcription factors have been identified in plants, including *Arabidopsis* (126) (Yanhui *et al.*, 2006), maize (157) (Du *et al.*, 2012a), rice (102) (Yanhui *et al.*, 2006), *Populus* (192) (Wilkins *et al.*, 2009), and cassava (166) (Liao *et al.*, 2016). The *M. truncatula* genome has been sequenced, yet the R2R3-MYB transcription factors have not been researched. This study performed genome-wide analysis of the R2R3-MYB transcription factors in *M. truncatula*. We identified 150 R2R3-MYB genes in *M. truncatula* and compared these with other plants. The R2R3-MYB genes of *M. truncatula* are similar to their *Arabidopsis*, maize, and cassava counterparts. The results indicated that R2R3-MYB transcription factors are highly conserved in plants.

Cannon *et al.* (2004) have shown gene duplication was closely related to plant evolution and played an important role in the gene amplification. Gene duplication analysis showed the same subfamily members were located on different chromosomes, such as the 21 subfamily gene members. These genes were distributed across all chromosomes and occurred as TD and SD. These results were consistent with the results of the phylogenetic tree. Furthermore, distribution of other subfamily members was confirmed based on clustering in the phylogenetic tree. These results suggest that the R2R3-MYB genes in *M. truncatula* are highly conserved as a result of gene duplication. The duplication pattern of R2R3-MYB genes, are consistent with the expression of R2R3-MYB genes in tomato (Zhao *et al.*, 2014) and *Populus* (Wilkins *et al.*, 2009). Tandem gene duplication was a major driver of gene expansion in *M. truncatula*.

MYB genes play a role in plant development and stress tolerance (Martin and Pazares, 1997). We have shown that R2R3-MYB genes were typically expressed in the roots, nodules, seedpods, and flowers. Compared with soybean and *Arabidopsis*, differential expression was observed during flower development, root formation, and seed development for R2R3-MYB genes (Du *et al.*, 2012b). Nodulation is the result of a symbiosis between legumes and rhizobial bacteria in soil. Libault *et al.* (2009) have reported a gene named Control of Nodule Development (CND), encoding an MYB transcription factor gene. When the CND gene is silenced, nodulation is reduced (Libault *et al.*, 2009). These results indicate that the MYB transcription factors may play a major role in regulation of legume-specific nodulation.

MYB transcription factor genes have been investigated as regulators for plant responses (Ambawat *et al.*, 2013). Their results show that MYB genes are involved in response to various abiotic stresses in higher plants. Herein, we found that MYB genes were not only down-regulated, but in some cases up-regulated in response to drought and salt stress. A positive response of MYB genes following drought stress, salt stress, and ABA-induced stress has been observed in *Arabidopsis* (*AtMYB002*, *AtMYB060/AtMYB094*, *AtMYB044*, and *AtMYB096*) (Cominelli *et al.*, 2005; Jung *et al.*, 2008; Seo *et al.*, 2009; Urao and Shinozaki, 1993), *Boea crassifolia* (*BcMYB1*) (Chen *et al.*, 2005), and *Saccharum officinarum* (*ScMYBAS1*) (Prabu and Prasad, 2012). However, MtMYB genes were up-regulated in response to cold and freezing stresses, while the opposite is observed in *Arabidopsis*. Interestingly, our result agrees with the expression profile of cotton in response to drought and salt stress (He *et al.*, 2016). Both cotton and *M. truncatula* are diploid and their duplication can be divided into TD and SD. The expression pattern of *M. truncatula* MYB genes under different abiotic stress conditions suggest that some may play a major role in cross-talk among different signal transduction pathways in response to abiotic stresses.

## CONCLUSIONS

In summary, we have identified 150 MYB genes in *M. truncatula*, which were classified into 21 subfamilies based on phylogenetic analysis. Meanwhile, their expression profiles were investigated using microarray and RNA-seq. The results revealed regulatory roles in plant growth and tissue development, and especially nodule development. In addition, we explored the role of the MYB genes in response to abiotic stresses. Our results suggested MYB transcription factors broadly participate in abiotic stress response of *M. truncatula*, whose function can be carefully explored in future.

## Supplementary material

The following online material is available for this article

Table S1 PCR primers used for quantitative real-time PCR analysis.

Table S2 Expression data of R2R3-MYB transcription factors in various tissues and development processes.

Table S3 Expression data of R2R3-MYB transcription factors in various tissues.

Table S4 Expression data of R2R3-MYB transcription factors response to abiotic stresses.

*Associate Editor: Hong Luo*
