# Supplementary material for: Genome-wide identification and characterization of R2R3-MYB genes in Medicago truncatula
Source: Genet Mol Biol. 2019 Nov 14;42(3):611–23. doi: 10.1590/1678-4685-GMB-2018-0235 (PMC6905446; doi:10.1590/1678-4685-GMB-2018-0235)
Supplement: Supplementary file 1 [file 1415-4757-GMB-42-3-2018-0235-suppl1.pdf]

Supplementary Material to "Genome-wide identification and characterization of R2R3-MYB genes in *Medicago truncatula*"

Table S1 - PCR primers used for quantitative real-time PCR analysis.

Gene Name	Forward Primer (5'to 3')	Reverse Primer (5'to 3')
MtMYB010	TGAAGTTGAAGAAGGAGAGAG	TGGCCATGTTTTTGGATGTA
MtMYB011	TGTGGAAAGAGTTGCAGATT	TTTTCTTCAAATGGGTGTGC
MtMYB012	TGCTCTTCCTAAACAAGCC	TGTTCGTCCTGGTAATCTTG
MtMYB013	TGCTTATGTCACCAGATACG	ACCATCTATTGCCAAGCTTT
MtMYB014	GACTGCTGAAGAAGATAGGA	TGACCATTTATTCCCAAGCT
MtMYB054	CCGAGGAAGACAAAAAGTTG	CATGGTCCATCTATTACCCA
MtMYB090	ATGGAGATGGTCATTGGAAC	TTCCGCTATTTTAGACCACC
MtMYB100	TGTTCCTCAGAAACAAACCA	GAAGAAAATGGACGGTGTTC
MtMYB108	CCAACACACACACAAACTTT	TTTTCAATCCTGTAAGCCGA
MtMYB116	AATGGGAAGACAACCTTGTT	GGCCTTAGGTAATTTGTCCA
GAPDH	TAAGGGTGGTGCCAAGAAGGT	AGCAAGAGGAGCAAGGCAGTT
