# Supplementary material for: Genome-wide identification and characterization of R2R3-MYB genes in Medicago truncatula
Source: Genet Mol Biol. 2019 Nov 14;42(3):611–23. doi: 10.1590/1678-4685-GMB-2018-0235 (PMC6905446; doi:10.1590/1678-4685-GMB-2018-0235)
Supplement: Supplementary file 2 [file 1415-4757-GMB-42-3-2018-0235-suppl2.pdf]

Supplementary Material to "Genome-wide identification and characterization of R2R3-MYB genes in *Medicago truncatula*"

Table S2 - Expression data of R2R3-MYB transcription factors in various tissues and development processes.

Gene	Leaf	Petiole	Buds	Stem	Flower	Pod	Seed10 DAP	Seed12 DAP	Seed16 DAP	Seed20 DAP	Seed24 DAP	Seed36 DAP	Root	DeNod 28DPI	Root0 DPI	Nod4DPI	Nod10 DPI	Nod14 DPI	Nod28 DPI	Nod16 DPINO3
MtMYB001	18.6695	9.22229	27.9771	10.6191	40.3587	12.7178	8.57797	8.29303	8.6647	23.536	35.3727	37.9749	7.47888	8.19284	9.76109	8.25064	8.36785	8.51868	8.11638	7.46877
MtMYB002	10.9841	46.1257	47.937	43.8326	230.499	10.1321	11.6613	13.4037	23.5574	49.6893	68.9137	152.071	6.32317	6.23872	6.13651	6.49154	6.4305	5.97794	6.49845	5.98999
MtMYB003	37.5789	234.808	140.825	112.975	379.777	72.0876	169.293	68.9112	36.3303	26.183	28.0642	204.628	6.16303	6.79428	6.84688	41.0759	33.7338	24.1305	18.7385	30.3795
MtMYB005	518.343	9.54989	194.501	8.20697	9.50902	122.993	6.75414	7.14773	7.46483	6.67792	7.48893	7.32674	7.95282	8.62225	7.12	7.14387	62.6968	17.0431	10.382	14.5759
MtMYB006	246.047	7.55387	94.5341	8.0092	8.0112	39.247	7.91855	7.32741	7.14006	8.54507	9.09711	8.82594	7.69859	7.1328	7.977	8.03508	11.4565	8.68614	8.38927	9.80232
MtMYB007	8.42796	9.84064	9.20127	9.56496	8.30265	10.0715	9.37754	9.40915	9.69652	9.64805	9.95633	9.92917	9.91449	61.8999	9.50531	17.4351	15.4862	11.3476	16.6602	12.7407
MtMYB008	5.56028	5.25471	5.35051	5.41139	5.23969	5.46108	5.22856	5.39282	5.40573	5.51277	5.47635	5.105	5.82903	5.58567	5.50943	5.62304	23.496	17.1863	6.93586	6.37035
MtMYB009	15.8097	15.2559	10.2287	16.7296	9.24432	7.28093	7.84206	7.21303	7.89641	7.95168	11.2399	67.4428	150.261	173.283	93.6951	75.3357	34.4166	66.3539	32.8209	213.977
MtMYB010	252.525	510.151	452.373	723.751	206.018	312.188	110.318	69.5724	105.555	488.68	779.166	208.454	1796.3	2401.32	2156.76	2568.53	1938.34	2148.19	1584.49	2895.38
MtMYB012	8.63356	8.72966	12.0379	14.1621	8.27103	7.27202	10.4755	7.84014	6.76254	6.61246	6.81172	10.1149	59.1585	43.0966	43.465	46.715	64.1689	96.7399	78.2292	155.27
MtMYB013	18.657	39.1499	88.6177	168	17.3193	12.6938	10.9161	10.4639	14.641	12.5509	11.381	10.1647	61.5217	1387.5	108.452	385.322	208.381	249.988	237.053	298.958
MtMYB014	11.8038	17.3841	51.4055	110.603	13.3535	11.2382	9.01375	9.96809	9.73864	9.73911	10.6174	15.474	12.9186	224.655	16.9673	68.7469	33.5483	36.2434	36.5579	33.7011
MtMYB017	322.774	376.599	551.92	367.166	1479.38	381.661	294.379	504.682	711.448	231.031	121.831	179.073	399.289	602.287	502.925	376.01	336.905	392.845	224.595	391.108
MtMYB019	13.6551	11.1386	8.96136	7.89073	217.991	12.1516	96.0269	56.4223	26.0477	36.8654	35.7358	14.464	6.95624	7.06924	10.2964	6.86929	7.52502	6.71239	9.16769	7.29089
MtMYB020	111.472	72.3175	102.959	104.116	312.624	144.663	180.446	146.213	94.9324	84.1376	65.1228	55.2222	276.838	379.634	254.728	178.135	314.071	240.108	171.913	396.478
MtMYB021	9.23582	10.0908	11.7376	7.74387	18.0859	18.9446	315.951	278.214	42.1613	7.68371	9.40713	37.2395	6.88869	7.35379	6.73156	6.73011	7.00051	7.03246	7.91144	6.54389

Gene	Leaf	Petiole	Buds	Stem	Flower	Pod	Seed10 DAP	Seed12 DAP	Seed16 DAP	Seed20 DAP	Seed24 DAP	Seed36 DAP	Root	DeNod 28DPI	Root0 DPI	Nod4DPI	Nod10 DPI	Nod14 DPI	Nod28 DPI	Nod16 DPINO3
MtMYB023	9.70695	8.30746	9.51519	8.25609	15.2946	14.7219	580.995	564.809	781.771	1127.61	978.052	178.967	7.23067	7.13191	7.53082	7.80381	6.92174	7.29265	7.99132	7.42126
MtMYB025	7.66437	7.55286	6.78226	7.55987	6.94245	8.77732	7.57264	7.81056	6.82242	8.02341	7.22947	7.34903	7.35198	6.69544	7.56421	6.39852	6.97588	7.29984	7.61293	7.1161
MtMYB041	10.2182	19.9425	25.0028	16.3171	21.2624	169.048	12.659	13.805	11.0916	17.3272	22.2775	672.395	540.087	344.061	336.059	437.81	531.678	466.956	30.3516	718.248
MtMYB045	85.9131	74.6288	324.973	80.0273	199.131	175.116	829.465	384.328	95.8435	82.9458	58.884	52.0225	7.12313	7.78845	7.85586	13.4639	10.9012	9.63229	16.7792	9.56009
MtMYB046	178.317	72.7542	151.341	76.6137	70.3761	42.0196	160.79	129.469	20.4791	35.9755	33.6161	64.5921	9.91698	11.0517	9.72373	8.46803	11.8329	9.21375	10.0898	9.93801
MtMYB047	8.78362	13.4025	19.5377	17.4665	23.8323	73.7253	17.399	13.2087	13.6172	11.5566	9.71481	7.52444	8.2881	8.07292	7.95687	8.46063	6.58259	7.15975	8.52142	7.93136
MtMYB049	9.56219	9.95345	10.2709	9.83872	8.49813	10.3014	9.52351	9.52214	10.0504	11.6326	9.86942	11.2771	11.1505	21.1586	12.3788	17.7815	10.3216	15.8142	13.7029	24.2486
MtMYB050	7.44757	6.58067	7.10659	6.24881	6.23967	7.39833	6.09539	8.09315	8.01256	7.2524	7.20493	6.85876	10.2722	17.2282	13.9641	20.6157	14.8384	21.4167	16.3825	23.4698
MtMYB051	12.9876	9.28661	11.9713	9.4497	8.87338	12.3916	9.64538	9.64217	11.3192	11.3833	9.87173	13.3632	129.469	261.033	128.409	64.8792	76.7395	113.45	71.5661	199.495
MtMYB054	7.2958	8.79242	31.468	28.8662	8.53979	50.558	6.73003	6.3257	7.61317	7.43818	6.73454	6.79765	6.53279	7.03706	7.18345	6.59837	8.19036	6.76828	7.42768	7.64706
MtMYB056	7.79971	7.53736	6.2631	7.37346	6.42736	6.52268	6.38856	6.55021	8.02874	6.6149	7.13905	7.79318	6.78687	7.04243	7.9057	6.70651	6.36315	6.61777	7.48186	6.76897
MtMYB057	15.6284	54.023	39.0667	111.601	21.4581	115.892	283.472	652.924	1172.76	634.835	397.968	33.5087	296.635	306.131	495.827	88.9131	20.0675	29.9675	15.5211	89.0788
MtMYB058	16.9489	437.199	75.7286	404.496	193.49	80.8748	51.7558	148.203	247.753	112.141	119.764	23.9402	16.1863	19.2997	28.0787	13.3181	11.3721	10.2347	8.64092	12.3048
MtMYB060	6.48795	8.95075	12.561	14.3811	6.84007	6.71992	6.5336	6.68666	6.8019	6.67195	6.52552	7.48912	9.53178	9.53379	20.1482	6.99063	7.35819	6.46694	6.59095	7.25156
MtMYB061	14.3016	16.2277	32.3108	44.9003	12.4341	111.394	47.9255	158.867	202.091	47.4035	29.1017	16.6263	23.964	39.4494	36.4022	17.4121	12.1089	12.5893	15.2092	11.6725
MtMYB062	17.6674	24.2955	29.1716	26.1583	13.0364	25.5959	64.938	241.364	416.126	110.272	31.2716	10.209	16.0298	13.6235	17.2206	12.4432	10.2514	9.25577	10.2738	10.8744
MtMYB065	18.3751	19.1575	43.1919	21.4214	52.3831	25.2969	17.878	22.9728	27.4204	36.0063	23.1024	20.938	252.622	364.165	213.49	441.544	487.493	436.502	481.169	429.43
MtMYB066	14.2161	13.9215	11.1183	12.84	9.71913	10.689	8.46205	10.2751	13.3723	10.972	11.8185	15.9767	92.5417	71.7303	43.23	212.759	153.766	111.675	81.9858	142.997
MtMYB069	8.99374	8.20428	8.23655	7.18837	8.49219	9.57331	9.44647	9.4931	8.34683	10.0865	8.37325	10.3515	175.84	103.592	146.51	48.9037	27.1588	42.3635	32.0477	61.4565

Gene	Leaf	Petiole	Buds	Stem	Flower	Pod	Seed10 DAP	Seed12 DAP	Seed16 DAP	Seed20 DAP	Seed24 DAP	Seed36 DAP	Root	DeNod 28DPI	Root0 DPI	Nod4DPI	Nod10 DPI	Nod14 DPI	Nod28 DPI	Nod16 DPINO3
MtMYB070	9.67114	9.11427	12.1812	8.23302	8.75336	8.33883	6.99358	8.99089	11.6555	10.2938	7.83155	6.61421	262.796	214.477	213.11	576.022	537.062	523.967	403.256	631.813
MtMYB071	15.0748	29.1923	17.5464	29.702	20.5789	15.2818	47.069	22.5078	18.0557	22.7911	26.9199	18.3525	322.8	435.862	417.75	147.573	109.453	149.798	121.061	260.134
MtMYB073	9.85255	9.3637	10.0942	11.559	11.1119	12.9324	16.8263	16.0136	22.87	19.223	14.0531	14.8923	319.112	521.739	811.49	589.607	252.274	296.038	131.275	316.891
MtMYB076	10.6819	11.7815	11.7977	10.7109	12.96	14.9624	14.2126	11.363	12.3821	12.9291	13.0707	13.2353	11.6141	13.9431	10.9067	11.8291	10.5209	15.1168	12.6314	10.4422
MtMYB077	7.22636	6.46434	6.29845	6.31307	7.01782	7.81993	7.55888	7.45691	7.93773	7.54176	8.34691	7.8505	6.97803	6.90956	7.19826	6.6991	7.01968	8.99419	7.02098	7.25043
MtMYB082	6.38819	6.94569	6.70505	6.85018	7.09591	7.2049	7.54667	7.38465	7.2491	6.95163	6.92146	7.12301	6.23546	6.84848	7.78837	6.50266	6.40931	6.95816	6.28726	6.01589
MtMYB087	9.58025	10.7942	9.82482	11.692	6287.34	10.9547	9.18858	10.5077	10.1523	12.1827	11.0339	10.6248	9.89769	9.94455	10.2952	10.2022	9.0549	8.8994	9.85527	9.94582
MtMYB088	9.56608	7.8577	7.28354	7.80596	5999.27	9.49349	7.4685	7.68112	7.8979	6.88981	8.10992	7.26866	7.87537	7.35252	7.56181	7.19148	7.18243	7.43494	7.54033	7.86119
MtMYB089	11.9301	12.1118	13.3433	11.3627	83.024	70.1257	18.2028	18.2055	24.779	84.2801	200.508	427.075	123.015	103.255	21.9427	65.2836	122.862	140.843	29.3211	534.016
MtMYB090	10.5187	9.52964	9.9948	11.8548	7.77042	9.22324	13.1904	10.3719	12.9356	11.4113	11.3331	10.5791	12.0001	180.893	9.90074	18.8014	19.246	19.2516	16.1519	91.9456
MtMYB091	6.85866	7.51171	6.74748	6.7857	14.0343	7.89084	6.95939	7.39062	7.24004	7.5153	7.48149	9.78788	6.60221	6.70088	6.9228	7.57016	7.24709	7.41235	7.81956	7.32438
MtMYB092	13.937	14.014	15.1299	13.3783	45.6787	15.0242	15.6437	20.2514	26.2054	60.3197	65.1027	60.6036	35.4041	65.2106	49.1664	57.7618	135.946	93.196	24.6241	372.174
MtMYB093	9.23641	8.62745	10.715	10.2092	18.8444	15.248	8.28412	8.78947	8.57322	8.94204	9.82264	9.90876	11.9945	9.3564	11.5831	9.70005	7.89685	9.01575	9.08096	8.38954
MtMYB094	11.4183	11.327	14.6345	11.6705	35.6682	23.1233	10.1569	7.24331	7.82839	10.6511	27.7044	72.1278	7.5619	7.98919	8.02188	8.75512	9.91511	8.62841	9.4796	7.42122
MtMYB099	12.7603	8.85291	19.2598	12.0808	22.5107	12.6124	15.01	15.2879	26.0371	45.8334	90.0088	17.4852	34.6761	12.7321	13.995	10.291	12.333	13.4323	13.5591	10.3934
MtMYB100	90.4908	78.745	341.414	511.27	176.244	286.699	234.67	77.3075	22.0343	37.559	134.309	172.586	527.003	2654.62	611.526	2008.11	1679.16	1835.88	2612.07	2441.05
MtMYB101	661.67	512.14	668.031	792.749	1980.03	623.379	679.192	601.193	478.681	808.154	1593.05	7100.5	671.414	1323.39	1070.32	1348.18	1429.95	1560.74	1501.29	2233.58
MtMYB102	1309.33	1873.68	2173.1	3133.72	2841.44	1296.64	1278.99	1471.36	1197.54	1236.68	1396.25	2892.52	2191.77	3430.24	2050.88	2319.39	2322.8	1862.82	2130.62	3248.93
MtMYB103	23.3155	23.1364	40.709	35.1995	19.7634	25.6015	26.6812	106.089	202.107	148.674	73.5327	20.1091	15.3605	24.5468	23.568	22.0496	9.52851	14.0944	17.1915	15.2055

Gene	Leaf	Petiole	Buds	Stem	Flower	Pod	Seed10 DAP	Seed12 DAP	Seed16 DAP	Seed20 DAP	Seed24 DAP	Seed36 DAP	Root	DeNod 28DPI	Root0 DPI	Nod4DPI	Nod10 DPI	Nod14 DPI	Nod28 DPI	Nod16 DPINO3
MtMYB105	22.4835	27.1715	51.9711	43.2578	63.8128	70.4847	169.667	141.381	161.65	133.385	134.858	191.981	70.5491	80.4221	41.8157	53.3851	95.288	68.6014	74.8461	71.7126
MtMYB106	28.8835	28.0512	25.7451	24.9455	25.2873	29.9006	43.6446	35.938	22.2935	25.6476	27.2949	24.852	113.721	466.599	483.245	492.265	88.1305	195.908	351.277	132.736
MtMYB107	311.667	274.997	1029.07	517.111	477.474	810.467	1426.08	1473.41	1075.86	818.004	764.89	549.254	533.086	639.698	925.216	1096.68	478.217	563.362	502.071	514.998
MtMYB108	9.37433	11.4572	9.15989	11.1104	10.8225	11.2507	11.6679	13.805	11.1204	9.33532	11.0292	10.2915	11.4905	13.4141	10.4738	17.5271	27.4515	25.9605	10.9623	66.0015
MtMYB111	10.0007	9.64382	9.89092	8.76243	10.7465	10.4118	11.0626	11.0724	11.7137	11.6328	10.4607	11.4858	11.1834	15.3746	28.5528	12.8919	10.8192	9.78601	9.98083	11.5483
MtMYB114	20.8059	42.0778	25.7347	71.3145	17.6284	30.2066	15.2579	12.9434	13.3842	19.1319	17.1228	17.0408	25.3811	31.4063	41.2901	25.9976	17.3628	20.5253	21.2716	18.6039
MtMYB115	7.33596	9.48272	8.12642	8.40915	9.39429	85.7723	8.18746	9.42093	8.43737	9.06117	8.46634	8.35742	9.62908	10.1452	11.3477	8.72029	8.65209	8.57768	8.59364	8.08576
MtMYB116	92.9091	50.1067	30.0124	39.5398	12.3124	15.9108	8.89237	9.8126	11.886	8.1256	9.37021	9.44861	29.5015	62.2213	49.1493	53.9243	16.5211	21.8894	17.0979	30.6937
MtMYB117	7.1992	7.62836	7.52401	7.18788	8.70526	6.22712	7.03114	8.16345	7.10374	7.65102	9.36706	8.42913	6.78526	7.28832	7.76621	7.187	7.5674	7.26452	8.21479	7.19143
MtMYB118	15.7539	25.2049	22.4719	17.1337	202.661	25.9974	439.02	453.225	360.164	260.283	203.518	69.4873	18.087	24.2113	33.9414	16.5242	16.4042	17.8262	20.0384	20.7458
MtMYB119	18.9792	29.4032	37.8103	27.8196	17.8199	20.8855	38.9743	43.6679	10.7922	8.2556	7.30999	8.12741	51.3498	110.309	275.515	244.342	132.758	122.322	136.382	99.1024
MtMYB120	23.9981	71.0805	395.463	170.283	201.98	359.844	559.706	507.378	271.782	127.556	68.2536	16.9916	123.985	121.184	215.439	215.69	38.6009	81.8317	73.0243	53.1575
MtMYB129	214.135	789.202	372.096	541.056	110.83	286.204	70.0457	44.7232	83.9239	89.1436	77.3587	26.4484	382.97	636.978	612.037	260.72	121.368	135.046	107.46	133.995
MtMYB133	7.458	6.64515	6.77794	6.67483	6.52619	7.0767	6.73537	6.78029	6.62544	7.67875	8.22103	9.10883	7.45476	7.81103	6.90566	6.02487	7.25787	6.6774	6.55551	6.76764
MtMYB141	63.7086	66.8727	83.2442	58.6793	72.3092	67.566	59.5478	43.9711	50.8261	63.1473	51.5477	90.8864	81.7051	69.0641	29.2479	44.5253	60.9721	39.7631	53.3755	60.4525
MtMYB145	11.0852	9.15975	9.97571	8.66812	14.802	148.034	9.80013	9.59791	9.71387	9.61251	8.09638	7.74992	9.64193	8.85327	9.25829	9.4257	11.3188	8.78429	10.1174	9.24886
MtMYB149	19.7715	50.3298	31.1274	68.1887	20.7725	26.6456	18.3793	14.9137	17.765	14.0477	16.4959	13.5615	29.4454	43.9566	42.4233	22.9333	14.0758	15.3589	15.7333	13.2065
