# Supplementary material for: Genome-wide identification and characterization of R2R3-MYB genes in Medicago truncatula
Source: Genet Mol Biol. 2019 Nov 14;42(3):611–23. doi: 10.1590/1678-4685-GMB-2018-0235 (PMC6905446; doi:10.1590/1678-4685-GMB-2018-0235)
Supplement: Supplementary file 3 [file 1415-4757-GMB-42-3-2018-0235-suppl3.pdf]

Supplementary Material to "Genome-wide identification and characterization of R2R3-MYB genes in *Medicago truncatula*"

Table S3 - Expression data of R2R3-MYB transcription factors in various tissues.

Gene	Root	Nodule	Blade	Bud	Seedpod	Flower
MtMYB001	0	0	2.02765	0.680286	0.611345	1.68239
MtMYB002	0	0	0.898864	0.828894	1.58637	22.6195
MtMYB003	0.086252	1.28641	2.0893	4.22462	3.041	14.3875
MtMYB004	0	0	10.5851	2.27846	3.07458	3.70375
MtMYB005	0	0.276657	7.19003	2.40345	0.783297	0.062945
MtMYB008	0.585246	2.74209	0.257666	0	0	0
MtMYB009	10.5755	4.48941	2.79997	0	0.611122	1.08898
MtMYB010	43.3765	45.1165	6.5389	6.79459	5.59833	4.21329
MtMYB013	2.76582	2.01709	0.7757	0.240184	0.199797	0.885257
MtMYB016	0.472579	2.78366	0	0	0	0
MtMYB017	7.78982	8.78157	13.0688	11.0287	13.9959	43.9131
MtMYB018	0.131313	0	4.33162	1.89045	0.272296	19.4046
MtMYB019	0	0	0.325629	0.38923	1.34882	13.1804
MtMYB020	14.4857	18.783	6.83846	6.26353	5.82235	21.0533
MtMYB021	0	0	2.78332	1.28519	5.47359	6.90457
MtMYB022	0	0.093851	0	0	2.9327	1.66408
MtMYB036	0	0	1.95047	0	0.247014	193.016
MtMYB038	0.087322	0	7.26931	13.7159	0.612146	0.181553
MtMYB039	0	0.105005	7.43248	13.8973	0	0
MtMYB041	25.5118	4.23576	0.438951	0	8.25423	0.486433
MtMYB044	1.79383	0.227355	0	2.49661	0	0.627221
MtMYB045	0.542079	1.27742	8.34573	7.92594	9.87375	10.0794
MtMYB046	0	0.146375	4.45577	3.28072	1.31618	3.48076
MtMYB047	0	0	0	0.97384	4.60297	1.12309
MtMYB048	0	0.110901	0.214325	3.96728	6.06784	3.91934
MtMYB049	0.751276	2.77725	0.615228	0.191654	0.166925	0
MtMYB051	10.9516	9.88902	0.53451	0.457656	0	0.191967
MtMYB054	0	0	0.171278	1.77667	3.46214	0.917953
MtMYB057	15.3503	0.146901	0.81137	1.4981	3.32467	1.46906
MtMYB058	0.746914	0.067494	1.97679	8.29489	3.54872	9.33714
MtMYB061	2.24767	0.347286	0.771675	1.17562	8.48389	0.429168
MtMYB065	6.78817	15.91	0.134359	0.507307	0.154263	1.10542
MtMYB066	8.84858	11.8677	0.138921	0.90887	0.165696	0

Gene	Root	Nodule	Blade	Bud	Seedpod	Flower
MtMYB067	4.09824	2.3658	0.139809	0	0.457789	0
MtMYB068	2.60998	12.6793	0	0	0	2.26719
MtMYB069	10.7119	5.54862	0	0	0	0
MtMYB070	5.73492	12.1205	0.149721	0	0	0
MtMYB071	6.07423	4.65244	0	0.189357	0.172236	0.875862
MtMYB073	10.3808	5.64283	0.138136	0	0.164401	0.077492
MtMYB074	2.33475	3.31623	0	0.305433	0	0
MtMYB087	0	0	0	0	0.696863	405.582
MtMYB088	0	0	0	0	0	552.306
MtMYB089	2.39192	0.879019	0.257727	0	1.95014	2.54076
MtMYB092	1.18187	1.47315	0.133269	0.432429	0.597296	2.40109
MtMYB094	0	0.190061	1.3356	1.03672	2.25691	3.52858
MtMYB098	3.39167	1.01401	2.58804	1.95851	4.48299	0.409611
MtMYB099	2.88421	0.34033	0	1.34568	0.801087	3.16828
MtMYB100	6.20663	22.997	1.4835	0	3.84562	1.25328
MtMYB101	27.7613	61.7121	38.8579	27.4697	33.7721	97.7758
MtMYB102	68.4495	70.4887	69.8158	41.8931	50.278	113.746
MtMYB105	4.43891	4.88604	0.917846	2.96827	6.46511	3.7855
MtMYB106	3.47714	8.52661	0	0	0.189944	0
MtMYB107	9.27372	6.4448	4.97447	15.8669	10.8324	7.71789
MtMYB113	47.5913	26.1409	95.586	11.579	0.84934	1.55196
MtMYB115	0.63568	0	0	0.448323	7.42198	0.76763
MtMYB116	1.92588	0.637701	5.27193	0.625265	0.390696	0.533628
MtMYB118	0.886772	0.559702	0.605999	0.877836	3.33753	11.433
MtMYB119	2.98	10.2182	1.7748	1.61923	2.11384	1.65624
MtMYB120	3.54295	1.10424	1.02045	9.59689	10.748	4.88205
MtMYB122	132.16	32.513	296.298	34.2321	1.89521	21.4707
MtMYB123	3.90082	1.16719	4.1848	2.17943	10.7183	2.58582
MtMYB125	1.20878	1.58466	1.74951	2.07424	5.19175	3.18776
MtMYB127	2.75149	0.570617	0.937481	1.03483	4.45884	1.25326
MtMYB129	8.23627	2.61979	4.13829	8.84615	6.72264	2.22812
MtMYB138	1.31761	3.56645	21.9146	53.1993	42.6547	33.4077
MtMYB145	0	0	0	0	4.11776	0.297233
MtMYB150	1.9581	1.65881	1.64707	2.31977	2.68908	1.49972
