# Supplementary material for: Genome-wide identification and characterization of R2R3-MYB genes in Medicago truncatula
Source: Genet Mol Biol. 2019 Nov 14;42(3):611–23. doi: 10.1590/1678-4685-GMB-2018-0235 (PMC6905446; doi:10.1590/1678-4685-GMB-2018-0235)
Supplement: Supplementary file 4 [file 1415-4757-GMB-42-3-2018-0235-suppl4.pdf]

Supplementary Material to “Genome-wide identification and characterization of R2R3-MYB genes in *Medicago truncatula*”

Table S4 - Expression data of R2R3-MYB transcription factors response to abiotic stresses.

Gene	Control	Cold	Freezing	Osmotic	Salt	ABA
MtMYB001	0.817877	4.3132	0.668525	2.12289	0.996294	1.26733
MtMYB002	10.7412	14.3792	5.93559	16.0315	15.9251	10.4358
MtMYB003	5.08682	7.97461	3.09251	4.77231	6.51028	3.62872
MtMYB004	8.81074	4.94204	3.69608	12.4893	12.9119	18.006
MtMYB007	4.22237	52.2045	49.2708	11.2819	6.57466	10.8634
MtMYB010	93.7593	112.047	185.364	84.2946	79.4086	63.2428
MtMYB011	7.5626	59.2823	60.1513	20.4599	9.93886	23.0664
MtMYB012	2.02465	4.12102	18.591	1.83415	0.98693	1.66003
MtMYB013	48.5036	243.668	145.157	108.891	90.1448	117.51
MtMYB014	39.5214	175.942	74.3598	87.2033	106.018	107.969
MtMYB015	4.19568	5.37716	4.40115	2.98823	5.57788	2.88936
MtMYB017	32.6837	33.2521	43.7615	26.4112	26.8573	28.985
MtMYB018	0.741054	1.38539	0.287048	0.299036	3.11127	1.69779
MtMYB020	11.9892	14.2123	27.6016	8.59743	8.8431	5.17145
MtMYB038	0	7.48192	5.26864	1.8806	0.238698	0.136499
MtMYB039	0.242237	4.42127	0.539541	0.402033	0	0
MtMYB041	6.18379	8.82375	15.4251	10.845	8.15588	2.11005
MtMYB044	0.97103	2.12781	0.800472	0.557341	0.562934	0.132672
MtMYB045	6.78203	10.1274	6.28775	6.60688	8.28811	6.7417
MtMYB046	1.09605	4.36249	1.61176	1.2519	1.15225	1.44056
MtMYB049	0.453682	0.94994	2.67149	0.765	0.697139	0.917525
MtMYB051	5.98348	5.45127	9.04284	5.17248	1.1216	0.969617
MtMYB052	0.736737	5.36553	2.9605	4.99732	3.63551	2.69001
MtMYB053	3.12147	5.29722	2.44675	17.3182	10.2475	8.97826
MtMYB054	0.323988	9.75337	2.82158	1.04546	0.587408	0.683709
MtMYB056	0.523582	1.25856	0.861705	3.4179	0.878682	1.49043
MtMYB057	4.7198	5.14264	2.12496	2.73987	2.92873	1.3467
MtMYB058	10.1	6.87354	2.97466	8.8723	15.6282	7.81946
MtMYB061	4.57382	5.83488	5.89237	3.79245	3.37095	1.43048
MtMYB062	1.78872	3.45225	0.899967	2.11932	1.59277	1.05912
MtMYB063	2.88868	1.68807	1.10905	1.54758	4.38392	1.68097
MtMYB065	3.4989	3.2429	5.18872	2.09236	2.35282	0.69109
MtMYB066	3.82766	5.31921	8.13513	6.23277	7.05801	3.78108

Gene	Control	Cold	Freezing	Osmotic	Salt	ABA
MtMYB068	3.47118	3.98564	7.70445	4.22624	4.98937	2.53453
MtMYB069	5.48237	4.17028	5.7464	2.19972	3.35547	1.09121
MtMYB071	12.371	18.9106	36.5806	9.90668	12.9841	7.75889
MtMYB073	4.77429	2.90355	6.54758	4.93385	3.11234	2.04183
MtMYB089	2.42644	10.9809	11.0647	13.163	8.47899	4.58661
MtMYB090	3.80353	20.2426	10.3667	12.3469	8.50236	4.45178
MtMYB092	2.81509	4.18728	5.69053	4.39422	3.36039	3.71055
MtMYB098	4.2997	3.71147	4.05062	4.06418	5.50904	2.63263
MtMYB099	1.68832	1.78497	2.4178	1.56801	2.85337	1.04873
MtMYB100	27.7786	109.853	95.3215	51.836	41.697	124.053
MtMYB101	64.4773	60.6748	154.662	72.1218	58.2256	176.011
MtMYB102	76.999	105.089	167.438	114.105	104.533	191.773
MtMYB105	2.84874	2.48776	3.09295	3.53948	5.42547	3.46785
MtMYB106	3.50973	2.23393	3.46442	2.64657	2.23847	1.47441
MtMYB107	8.859	11.9294	12.7574	9.14922	8.41657	5.7219
MtMYB108	3.91675	10.804	28.7694	8.01797	4.33055	3.05778
MtMYB111	0.200846	0.127988	0.618352	2.56167	0.3935	2.09569
MtMYB113	100.859	84.5923	141.091	77.4123	92.5014	93.998
MtMYB116	5.41636	55.1172	18.1375	14.4414	14.5555	16.3256
MtMYB119	3.4995	4.50887	11.1403	3.02439	4.15602	2.44017
MtMYB120	2.02651	2.73775	2.22647	1.22962	3.0343	0.431797
MtMYB122	242.284	53.5986	150.998	148.929	147.043	141.503
MtMYB123	8.09602	7.06306	2.79621	9.11425	9.27705	5.25261
MtMYB125	6.26454	4.30647	3.27754	5.0624	5.30412	3.3897
MtMYB127	5.48587	7.42561	6.05446	6.29415	7.60622	4.41144
MtMYB129	15.5291	20.2115	14.4367	13.764	19.1948	8.64145
MtMYB132	1.77868	3.0433	3.39687	1.71048	0.892634	0.810569
MtMYB137	2.29551	1.63588	1.9617	2.08367	2.10463	2.02878
MtMYB138	15.94	9.69741	10.1352	17.3228	26.8352	23.2394
MtMYB149	2.83132	5.71508	3.17077	3.10375	4.27561	2.04922
MtMYB150	1.82464	1.55381	1.63308	2.18456	2.10583	2.89557
